# Selections

**Published:** 1851-07-01

**Authors:** 


### Selections.

Mortality in Lunatic Asylums.—In a communication on "The Health of London during the six months terminating March 29th, 1851," published in the "London Journal of Medicine," Dr. Webster states that the total number of deaths in establishments for insane patients, during that time, amounted to 221; of whom 116 were male, and 105 female, lunatics. This, he says, appears to be a large mortality, especially if contrasted with the previous summer and autumn quarters, when the aggregate deaths in the same institutions were 171. The results now mentioned are, however, consistent with general experience; since death more frequently supervenes amongst lunatic patients during the cold and severe weather of winter than in temperate seasons; notwithstanding that mental disease more generally attacks individuals in the hot days of summer than in the colder months. The comparative amount of fatal cases met with in the two sexes of lunatics is also interesting, as the figures quoted fully bear out a remark made by Dr. Webster elsewhere, namely, that "mania, although more common among women, is in them more curable, and less fatal than among men." A larger number of female lunatics being usually under treatment in the metropolitan asylum than of males, the occurrence of 105 deaths among the former sex, in contrast to 116 among the latter, strongly supports this view; especially if the facts recorded in the last report be remembered, wherein it is stated, that 96 insane male patients and only 75 females died during the period therein included. According to the above data, besides others which might be quoted, if necessary, a more unfavourable prognosis of the termination in cases of insanity may be given, *cæteris paribus,* in men than in women; whilst a greater number of recoveries may be confidently expected in the latter than in the former class of lunatics.

Ramollissement of the Brain.—Mr. W. F. Barlow, at a meeting of the Medical Society of London, narrated the particulars of a case of ramollissement of the brain, that had been under treatment at the Westminster Hospital, under the care of Dr. Roe. The patient, a man, was admitted on the 13th November, 1848. He had a heavy expression, and complained of pain in the head, at times more severe than at others; also of giddiness, and a difficulty of moving the right hand and fingers. His handwriting was like that of a person with chorea, and he occasionally let things drop. The memory was defective, and soon began obviously to fail; the processes of the reasoning faculties were altogether impaired. After the lapse of five days, during which the symptoms progressed somewhat, he changed abruptly for the worse, and lay in a state of stupor. This was not uniform, and he could at times be aroused to passing consciousness. There was complete paralysis of the right side, the leg being readily excited to reflex actions. He began to swallow, and also to breathe with difficulty, but, occasionally, the swallowing and breathing seemed unembarrassed. The vacillation of the symptoms formed one of the more interesting features of the case; now there was consciousness, now almost absolute loss of it; one who might have watched him superficially, or who might have judged of his condition by a single visit, would have been deceived. On the 22nd, he could not be roused so as to know anything, and both swallowed and breathed with difficulty. The paralyzed side was lax, and never affected by any spasms save those induced by reflex action; the other was almost continually agitated by aimless, restless acts of volition. The patient lingered till the 26th, and then died in deep coma.

On examination of the body after death there was found considerable opacity of the arachnoid, the vessels of which were more turgid and crowded than usual. But the main thing, was a most marked and extensive softening of almost the entire cerebral lobe of the left side. Small dots of blood were effused here and there throughout the softened brain. Some spots were of a pink or red hue, a yellow or yellowish tinge was seen nowhere. The inner portion of the brain was much more affected than the outer. The softening was extremely extensive and well marked.

Mr. Barlow, in commenting on the case, remarked upon general and partial ramollissement; the former was often a post-mortem change; the latter was far to be relied on as the consequence of disease. He next alluded to some of the symptoms marking the case, as the pain and vertigo, the imperfection of motion, the paralysis, the reflex action, the difficult breathing and deglutition, the variability of the important signs, the state of the mind throughout, and particularly on the absence of rigidity; a matter upon which he thought Lallemand laid infinitely too much stress in the diagnosis of ramollissement. Whether or no much pain would attend this affection would depend on the acuteness, violence, and extent of inflammation, and on the age and temperament of the patient; slight and very gradual inflammatory action would produce but little; and the coma which sometimes attended the affection would, of course, altogether preclude it. In those cases where the parts died, as it were, not violently, but lost their coherence from the effects of atrophy, pain was not to be expected, as in those instances where inflammatory action ran high. It was to be considered too, with reference to increased sensibility, whether the ramollissement were pure and uncomplicated, or combined with other affections—arachnitis, for instance. The pain of the disease should also be compared with that which happened under various circumstances; it was by contrasting symptoms that their nature was found out. The subject of vertigo was one of great moment in reference to cerebral affections in general; it was well marked in this instance: it happened sometimes from cerebral anæmia, at others, from dangerous fulness, and might be a threatening of apoplexy; at one while it was a mere sign of functional disorder, at others a most clear and alarming evidence of organic disease. It was one of the best marked and most constant symptoms in the instance of an enormous aneurism, lately presented to the Pathological Society by Dr. Roe. The way in which volition was affected deserved attention. By watching this function, the true state of the mind might almost be ascertained, and the progress of the case correctly estimated. As it became more influenced, there was an increase of the weakness of the mind, whilst, with the confirmed hemiplegia, came a state of all but perfect annihilation of the cerebral functions, and of danger most imminent. The variation in the symptoms towards the close of the case was one of the most remarkable features; life did not ebb gradually away; there were partial recoveries from time to time. The same thing had been noticed in various head affections; the sensation, the will, even the vital functions, most strangely differed during their course. To what is to be attributed that striking and complete recovery of consciousness just before death, in some cases of profound coma. To this question Mr. Barlow does not offer any adequate reply, merely putting it as a query, whether the brain is less compressed for a while, owing to a change in the cerebral circulation; he then proceeds to comment on the extreme difficulty of accurately investigating the condition of the faculties in cerebral affections, and on the unsatisfactory statements made by some writers of their being unimpaired in certain instances, whereas the non-recognition of their impaired condition was owing to the superficial and inadequate nature of the examination. The absence of rigidity or contraction of the limbs in the case under notice, Mr. Barlow considered well worth noticing, because of the stress laid by some authors on that form of muscular action, as a sign of ramollissement. It was anything but an indication of that process, although the frequency wherewith it happened in instances of that affection made its consideration, in reference to the diagnosis, very important. Taken in reference to cerebral diseases in general, it was of great importance to observe it well. It might arise, first, from physical irritation of the spinal cord; secondly, from the influence of emotion, as may be ascertained by examining the body carefully under various states; thirdly, from an affection of the muscles themselves, being the manifestation, probably, of an exhausted irritability. Fatty degeneration, in its relation to ramollissement and apoplexy, was next considered. A man, forty-one years of age, died in the Westminster Hospital, having an immense sanguineous effusion, chiefly occupying the left ventricle of the brain, and the contiguous cerebral substance. The smaller vessels of the softened substance were examined microscopically by Dr. C. Shearman, who found them in that state of fatty degeneration which Mr. Paget has described and figured in the *Medical Gazette*. The degeneration of vessels of that minute size was of great consequence; there could hardly be a doubt but that it led both to ramollissement and to apoplectic effusion.—*Medical Times.*

Hydrophobia.—Dr. Redfern, of Aberdeen, relates in the *Edinburgh Monthly Journal*, a case of hydrophobia occurring twenty days after the bite of the rabid dog, and terminating fatally on the fifth day. No structural lesion could be detected after death, even on the most careful examination.

